# Supplementary material for: Effect of Deterpenated Origanum majorana L. Essential Oil on the Physicochemical and Biological Properties of Chitosan/β-Chitin Nanofibers Nanocomposite Films
Source: Polymers (Basel). 2021 May 7;13(9):1507. doi: 10.3390/polym13091507 (PMC8124804; doi:10.3390/polym13091507)
Supplement: Supplementary file 1 [file polymers-13-01507-s001.zip › polymers-1204339-supplementary.pdf]

polymers

Supplementary Information

Effect of Deterpenated *Origanum majorana* L. Essential Oil on the Physicochemical and Biological Properties of Chitosan/β-Chitin Nanofibers Nanocomposite Films

Rut Fernández-Marín [1,*], Muhammad Mujtaba [2], Demet Cansaran-Duman [2], Ghada Ben Salha [1], Mª Ángeles Andrés Sánchez [1], Jalel Labidi [1] and Susana C. M. Fernandes [3,*]

1 Environmental and Chemical Engineering Department, University of the Basque Country UPV/EHU, Plaza Europa 1, 20018 Donostia-San Sebastián, Spain; bensalhaghadaangle@gmail.com (G.B.S.); marian.andres@ehu.eus (M.Á.A.S.); jalel.labidi@ehu.eus (J.L.)
2 Institute of Biotechnology, Ankara University, 06110 Ankara, Turkey; mujtaba@ankara.edu.tr, muhammadmujtaba443@gmail.com (M.M.); dcansaran@gmail.com(D.C.-D.)
3 IPREM, CNRS, Universite de Pau et des Pays de l'Adour, E2S UPPA, 64000 Pau, France
* Correspondence: rut.fernandez@ehu.eus (R.F.-M.); susana.fernandes@univ-pau.fr (S.C.M.F.)

Citation: Fernández-Marín, R.; Mujtaba, M.; Duman, D.C.; Salha, G.B.; Sánchez, M.Á.A.; Labidi, J.; Fernandes, S.C.M. Effect of Deterpenated *Origanum majorana* L. Essential Oil on the Physicochemical and Biological Properties of Chitosan/β-chitin Nanofibers Nanocomposite Films. *Polymers* **2021**, *13*, 1507. https://doi.org/10.3390/polym13091507

Academic Editor: Luminita Marin

Received: 15 April 2021
Accepted: 4 May 2021
Published: 7 May 2021

Publisher's Note: MDPI stays neutral with regard to jurisdictional claims in published maps and institutional affiliations.

Copyright: © 2021 by the authors. Licensee MDPI, Basel, Switzerland. This article is an open access article distributed under the terms and conditions of the Creative Commons Attribution (CC BY) license (http://creativecommons.org/licenses/by/4.0/).

S1. ^{13}C NMR of the β-Chitin Nanofibres.

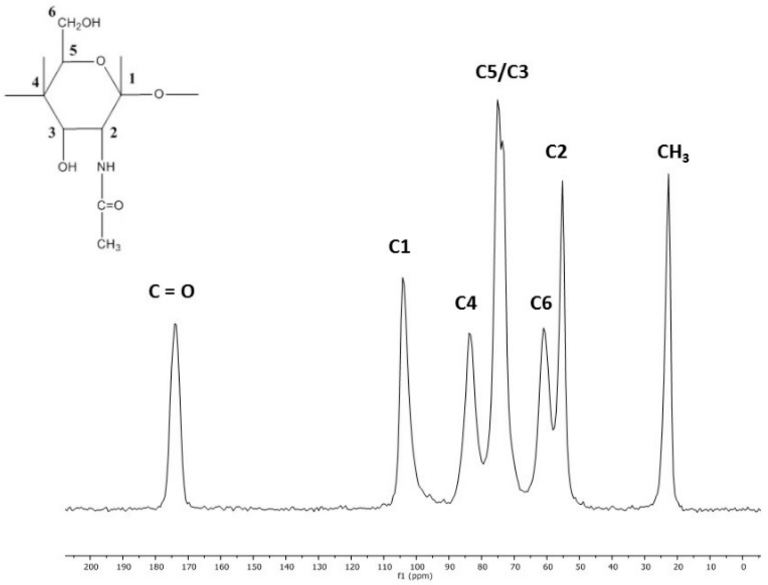

Figure S1. ^{13}C-NMR spectra of β-chitin nanofibres.

S2. Morphology of the β-Chitin Nanofibres.

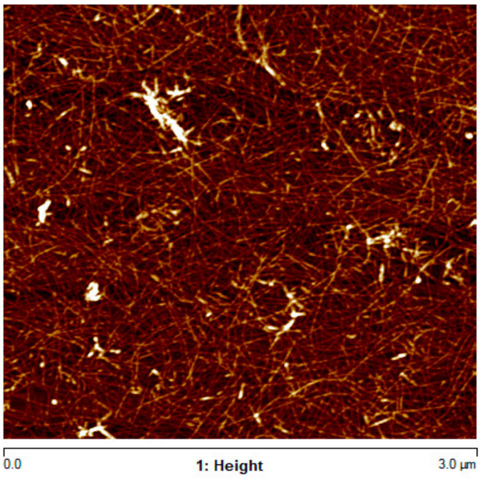

Figure S2. AFM images of β-chitin nanofibres.

S3. Cytotoxicity Assay

Acridine Orange (AO) Staining

The cells were cultured and were fixed with 4% PFA (paraformaldehyde) for 15 min. After fixation, the cells were stained AO for 30 min and the stained cells were analyzed with the Zoe Cell Imaging System (Biorad, USA).

DAPI Staining

Cell nuclear morphology was evaluated by fluorescence microscopy following DAPI staining. L929 cells were fixed with ice-cold 70% ethanol and resuspended in DAPI, and incubated for 15 min at 37°C wrapped in aluminum foil. The cells were then washed with PBS and examined under Zoe Cell Imaging System (Biorad, USA).

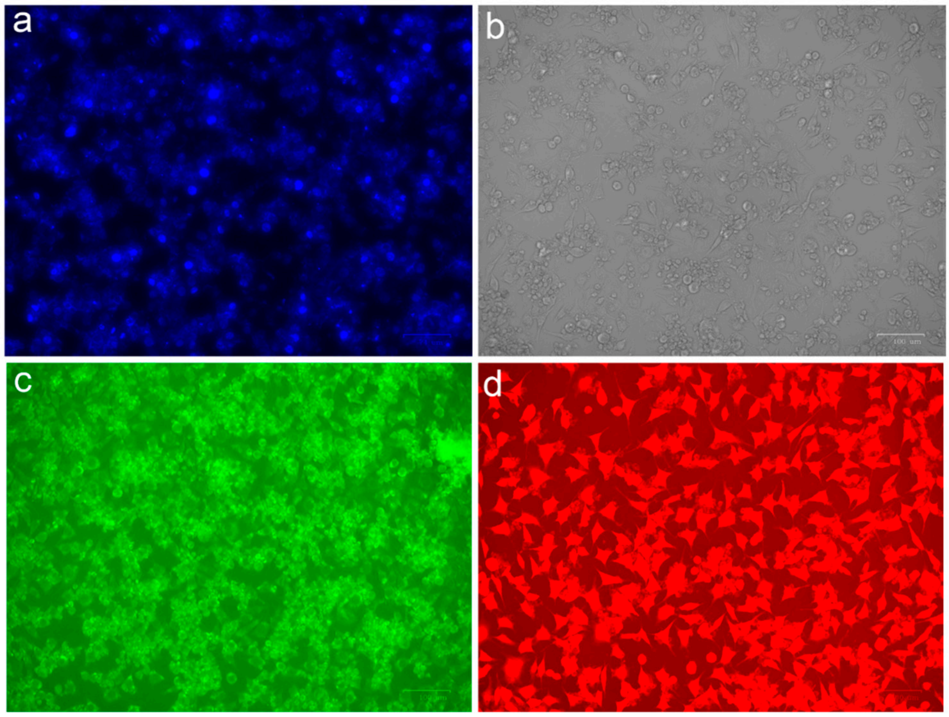

Figure S3. a and c) L929 cells by DAPPI staining; b) L929 cells using light microscopy; d) L929 cells using by acridine orange (AO) staining.

S4. Antifungal Activity

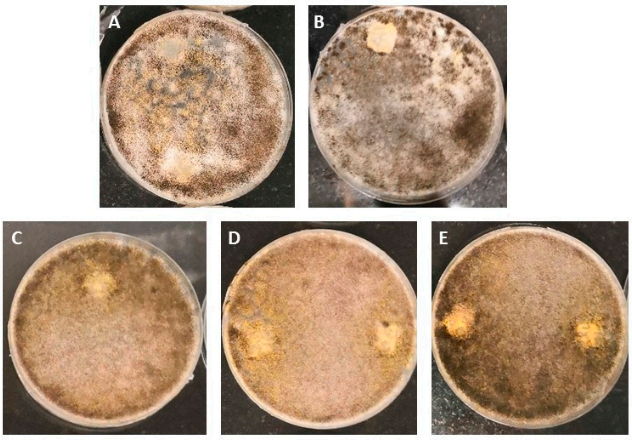

Figure S4. The general aspect after 7 days of incubation against Aspergillus niger. A) CSNF; B) CSNF-F1; C) CSNF-F2; D) CSNF-F3 and E) CSNF-OM.
